# Detection of potential drug-drug interactions for risk of acute kidney injury: a population-based case-control study using interpretable machine-learning models

**DOI:** 10.3389/fphar.2023.1176096

**Published:** 2023-05-23

**Authors:** Hayato Akimoto, Takashi Hayakawa, Takuya Nagashima, Kimino Minagawa, Yasuo Takahashi, Satoshi Asai

**Affiliations:** ^1^ Division of Pharmacology, Department of Biomedical Sciences, Nihon University School of Medicine, Itabashi-ku, Tokyo, Japan; ^2^ Division of Genomic Epidemiology and Clinical Trials, Clinical Trials Research Center, Nihon University School of Medicine, Itabashi-ku, Tokyo, Japan

**Keywords:** drug-drug interaction (DDI), machine learning, artificial inteligence, relative excess risk due to interaction, nephrotoxic drugs, acute kidney injury

## Abstract

**Background:** Acute kidney injury (AKI), with an increase in serum creatinine, is a common adverse drug event. Although various clinical studies have investigated whether a combination of two nephrotoxic drugs has an increased risk of AKI using traditional statistical models such as multivariable logistic regression (MLR), the evaluation metrics have not been evaluated despite the fact that traditional statistical models may over-fit the data. The aim of the present study was to detect drug-drug interactions with an increased risk of AKI by interpreting machine-learning models to avoid overfitting.

**Methods:** We developed six machine-learning models trained using electronic medical records: MLR, logistic least absolute shrinkage and selection operator regression (LLR), random forest, extreme gradient boosting (XGB) tree, and two support vector machine models (kernel = linear function and radial basis function). In order to detect drug-drug interactions, the XGB and LLR models that showed good predictive performance were interpreted by SHapley Additive exPlanations (SHAP) and relative excess risk due to interaction (RERI), respectively.

**Results:** Among approximately 2.5 million patients, 65,667 patients were extracted from the electronic medical records, and assigned to case (*N* = 5,319) and control (*N* = 60,348) groups. In the XGB model, a combination of loop diuretic and histamine H_2_ blocker [mean (|SHAP|) = 0.011] was identified as a relatively important risk factor for AKI. The combination of loop diuretic and H_2_ blocker showed a significant synergistic interaction on an additive scale (RERI 1.289, 95% confidence interval 0.226–5.591) also in the LLR model.

**Conclusion:** The present population-based case-control study using interpretable machine-learning models suggested that although the relative importance of the individual and combined effects of loop diuretics and H_2_ blockers is lower than that of well-known risk factors such as older age and sex, concomitant use of a loop diuretic and histamine H_2_ blocker is associated with increased risk of AKI.

## 1 Introduction

Acute kidney injury (AKI) is one of four phenotypes of drug-induced kidney disease (DIKD), and is diagnosed by serum creatinine (SCr)-based definitions proposed in the Kidney Disease: Improving Global Outcomes (KDIGO) 2012 guidelines (Mehta et al., 2015; Ostermann et al., 2020). Various clinical studies have been conducted to assess the effect of individual drugs (e.g., platinum-based agents and antibiotics) on risk of AKI, and those drugs associated with increased risk of AKI are listed as nephrotoxic drugs (Usui et al., 2016; Huang et al., 2022). Recently, Gray et al. (2022) have classified the nephrotoxic potential of 167 medications into seven phased nephrotoxicity categories (ranging from “No potential” to “Definite”), and 41 medications (25%) had nephrotoxic potential (rating ≥ 1). In Japan, society is aging rapidly because of the declining birth rate, and while individuals aged 20–34 account for 4.9% of total cases of polypharmacy, individuals aged 65 and older account for 69.0% (Onoue et al., 2018). Therefore, it is possible that multiple drugs with nephrotoxic potential are prescribed to patients, especially elderly patients. In fact, it has been reported that in elderly patients, concomitant use of two drug classes with nephrotoxic potential, for example, antibiotics and proton pump inhibitors, is the 3rd leading cause of acute interstitial nephritis (AIN), which is an important cause of AKI (Muriithi et al., 2015; Pierson-Marchandise et al., 2017). Hence, it is important to evaluate the combined effect of two drug classes on the risk of AKI.

In 2000, a case report of two patients who had taken a diuretic, angiotensin receptor blocker, and non-steroidal anti-inflammatory drug (NSAID) in combination, so-called “triple whammy,” and experienced a rise in SCr was published (Thomas, 2000). Subsequently, a number of clinical studies worldwide have investigated whether concurrent use of these drug classes increases SCr level and decreases estimated glomerular filtration rate (Loboz and Shenfield, 2005; Lapi et al., 2013; Camin et al., 2015; Kunitsu et al., 2019; Imai et al., 2022). Besides the triple whammy, clinical studies have tried to detect drug-drug interactions between two or more drug classes in acute kidney injury (Bird et al., 2013; Gandhi et al., 2013; Gul et al., 2016; Inaba et al., 2019; Okada et al., 2019; Liu et al., 2021; Salerno et al., 2021). However, most of these studies used a multivariable logistic regression (MLR) model, which is a traditional statistical model, and evaluation metrics such as discrimination, calibration, and robustness of the regression model have not been evaluated. Machine learning (ML) is an alternative analytical approach that can handle complex relationships between a number of variables in real-world big data. ML algorithms have been used to predict AKI, and the predictive performance of ML models is often superior to that of traditional statistical models (Song et al., 2021; Peng et al., 2022; Yue et al., 2022). Furthermore, interpretable ML has been applied to detect variables affecting the development of an outcome (Jiang et al., 2023). Hence, the aim of the present study was to investigate whether there is a combination of two drug classes that has combined effects on the increased risk of AKI, by mining electronic medical records using interpretable ML models.

## 2 Materials and methods

### 2.1 Data source

The present study was based on a population-based case-control study utilizing electronic medical records from the Nihon University School of Medicine’s Clinical Data Warehouse (NUSM’s CDW) between 1 April 2004 and 1 September 2021. NUSM’s CDW is a centralized data repository that integrates separate databases, including patient demographics, diagnoses, and laboratory data of approximately 2.5 million patients, from the hospital information systems at three hospitals affiliated with the NUSM; Nihon University Itabashi Hospital, Nerima Hikarigaoka Hospital, and Surugadai Nihon University Hospital. To protect patient privacy, patient identifiers are replaced by anonymous identifiers in all databases of the CDW.

### 2.2 Definition of acute kidney injury as binary outcome

**FIGURE 1:**
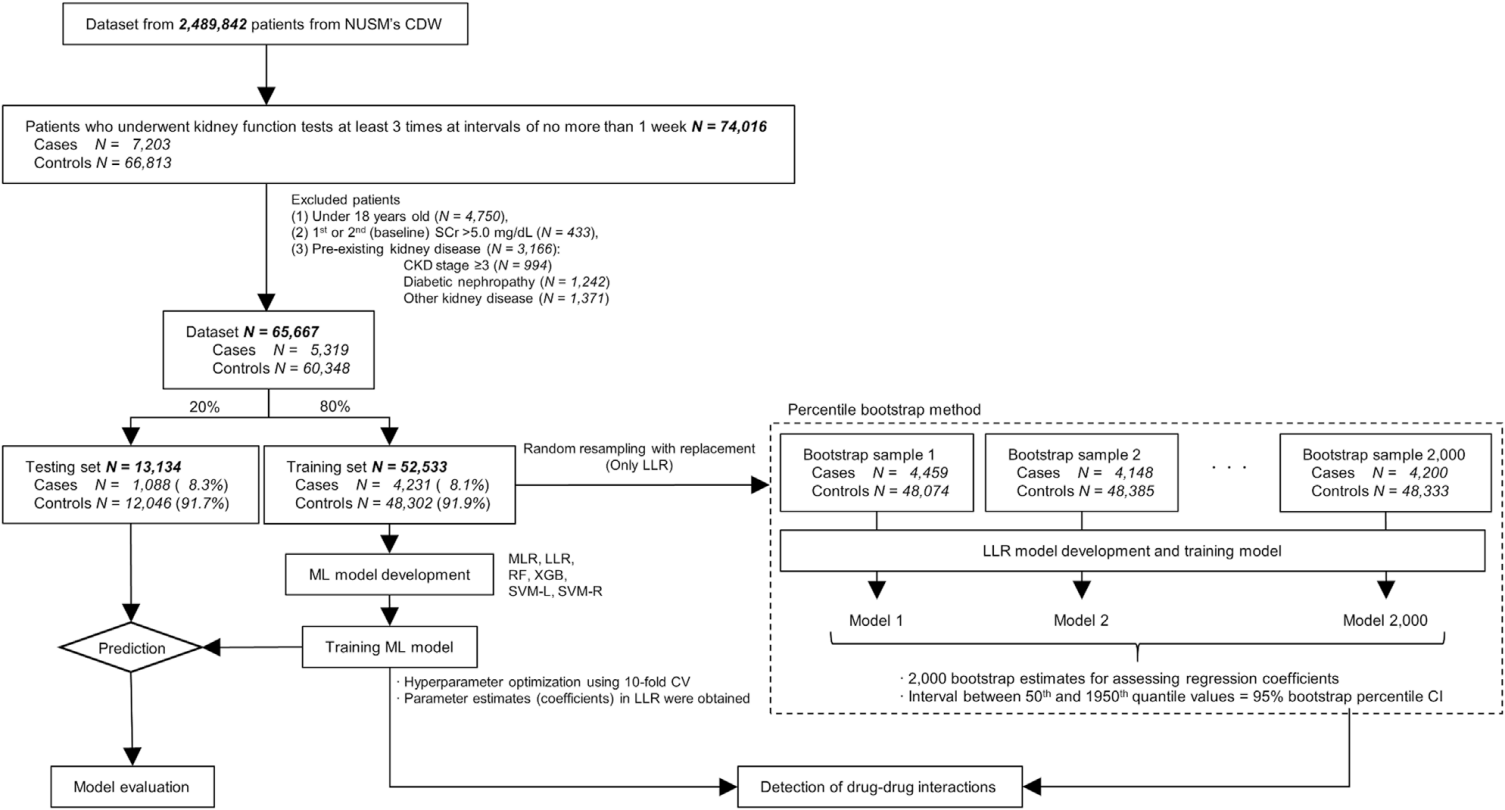
Sample size flowchart. Abbreviations: NUSM’s CDW, Nihon University School of Medicine’s Clinical Data Warehouse; SCr, serum creatinine; CKD, chronic kidney disease; CI, confidence interval; LLR, logistic least absolute shrinkage and selection operator regression; CV, cross-validation; ML, machine-learning; MLR, multivariable logistic regression; RF, random forest; SVM-L, support vector machine (linear function); SVM-R, support vector machine (radial basis function); XGB, extreme gradient boosting.

Sample size flow in this study is shown in Figure 1. Firstly, 74,016 Japanese patients who underwent kidney function tests at least three times within 14 days (the interval between each measurement date was 7 days or less) and whose serum creatinine (SCr) showed a <50% change between the 1st and 2nd measurement dates were extracted from NUSM’s CDW, and the 2nd measurement date was regarded as baseline. Among the 74,016 patients, those who met any of the following two conditions were regarded as patients with acute kidney injury complying with the KDIGO criteria: 1) SCr increased by 0.3 mg/dL within 48 h from baseline, or 2) SCr increased to ≥1.5 times higher than baseline within the prior 7 days. These patients with acute kidney injury were assigned to the case group (*N* = 7,203; outcome = 1) and the date that AKI occurred was regarded as the event date. On the other hand, the remaining 66,813 patients were assigned to the control group (outcome = 0), and the 3rd measurement date was regarded as the reference date in the control group. Next, patients who met the following exclusion criteria were excluded: 1) under 18 years old, 2) baseline SCr >5.0 mg/dL, and 3) patients with pre-existing kidney disease [chronic kidney disease stage ≥3, diabetic nephropathy, other kidney disease; International Classification of Disease 10 (ICD-10) codes are shown in Supplementary Table S1A]. Then the clinical information from 65,667 patients was used for training and testing of ML models.

### 2.3 Features

In order to detect interactions between two drug classes for risk of AKI, we obtained use or non-use of 32 therapeutic drug classes, and 496 (=32C2) product terms of these drug classes as features from the eligible patients. However, because none of the 88 product terms included patients who developed AKI (i.e., these product terms contained only “0”), the number of product terms reduced to 408. The drug classes were classified based on the Anatomical Therapeutic Chemical (ATC) code published by the WHO Collaborating Centre for Drug Statistics Methodology (Supplementary Table S2). AKI tends to occur within 7 days from the initiation of a culprit drug, and sub-acute kidney injury occurs within 4 weeks (Mehta et al., 2015). In fact, it has been reported that several nephrotoxic drugs are more likely to induce acute kidney injury within 7 days, and most cases of acute kidney injury occur within 30 days from the initiation of the drug (Khalili et al., 2013; Miano et al., 2018; Ide et al., 2019; Kunitsu et al., 2022; Wu et al., 2022). Hence, if a drug class was newly started within 1–7 days before the event date, the drug class was regarded as “use.” If a drug class was newly started on the event date, the drug class was regarded as “non-use,” to prevent reverse causality bias. A drug class newly started within 8+ days before the event date was regarded as “non-use.” When the included product term is “1,” it means that the two drug classes were newly started at about the same time within 7 days before the event date, whereas when it is “0,” it means that one of the two drug classes was used or neither of them was newly started. That is, the present study detected whether any of the 408 combinations of the 32 drug classes had an interaction for increased risk of AKI.

Five demographic characteristics and medical history which included seven diagnoses as features were obtained to adjust for the effect of these features on the risk of AKI. The demographic information was composed of age, sex, and three hospitals (Itabashi, Hikarigaoka, and Surugadai; dummy variables). The medical history was composed of hypertension, diabetes, heart failure, anemia, sepsis, chronic kidney disease (stage ≤2), and chronic liver disease, which are known risk factors for AKI (ICD-10 codes are shown in Supplementary Table S1B) (Poston and Koyner, 2019; Ostermann et al., 2020; Yu et al., 2020; Cullaro et al., 2022). A diagnosis was regarded as “present” if there was a diagnosis before baseline. We investigated 32 therapeutic drug classes commonly associated with risk of acute kidney injury (Usui et al., 2016; Nast, 2017; Ostermann et al., 2020). Finally, a two-dimensional dataset (65,667 patients × 452 features) for ML was generated. Data imputation was not performed because all the observations in the dataset had no missing values.

### 2.4 Construction of ML models and model evaluation

The dataset for ML was randomly split into a training set for the development of ML models (80%; *N* = 52,533) and a testing set for evaluation (20%; *N* = 13,134). To evaluate the effects of individual drug classes and their product terms on the risk of AKI, six ML models were utilized in this study: 1) MLR model and 2) logistic least absolute shrinkage and selection operator regression (LLR) model which are linear algorithms, 3) random forest (RF) model and 4) extreme gradient boosting (XGB) tree model which are tree-based algorithms, and 5) and 6) two support vector machine models [kernel = linear function (SVM-L) and radial basis function (SVM-R)]. All the supervised ML models were developed using R software (version 4.1.2; R Foundation for Statistical Computing, Vienna, Austria).

The supervised ML models were performed with AKI occurring as a binary dependent variable and the 452 features as independent variables; that is, 449 features excluding the individual effects of the two drug classes of interest and their product terms were regarded as co-variables. When constructing the four ML models except for the MLR model, we ran 10-fold cross-validation to perform hyperparameter tuning. In the LLR model, a lambda (*λ*) value, which is the penalty term in the loss function, was determined to minimize misclassification error for the training set and to avoid over-fitting to the training set using the R “glmnet” package. A regularized logistic regression equation was obtained using the optimized *λ* value (Supplementary Figure S1). The RF model was constructed using the R “randomForest” package. Hyperparameters such as the number of features randomly sampled as candidates at each tree (mtry) and the number of trees to grow (ntree) were optimized by grid search (Supplementary Table S3). The XGB model was constructed using the R “xgboost” package. The hyperparameters of the XGB model are roughly divided into the following four parameters: general, booster, learning task, and command line parameters. Of these parameters, booster parameters were optimized by grid search. Finally, the XGB model with the optimized hyperparameters was constructed (Supplementary Table S4). The two SVM models were constructed using the R “e1071” package, and the hyperparameters were optimized using a grid search (Supplementary Table S5).

Area under the receiver operating characteristic curve (AUROC) and area under the precision-recall curve (AUPR) were calculated to evaluate the discrimination and robustness of each ML model. To evaluate model calibration, the calibration slope and intercept were calculated for each ML model from a calibration plot with actual probabilities on the *X*-axis and log odds on the *Y*-axis. The calibration slope and intercept have target values of 1 and 0, respectively. A slope <1 indicates that predictive risk is too extreme, i.e., too high for patients who are at high risk and too low for patients who are at low risk, and an intercept <0 indicates overestimation of predicted risk (Van Calster et al., 2019). Additionally, Brier score, which is an evaluation metric to verify the accuracy of predicted probabilities, was calculated for model calibration using the R “scoring” package. Brier score is the mean squared error between the actual binary outcome and the predicted probabilities, as shown in Formula 1 (Huang et al., 2020):

$$Brier\;score=\frac{\sum_{i=1}^{N}\left(E_{i}-O_{i}\right)}{N}$$ (1)

where N is the number of patients, $E_{i}$ is the predicted probability for patient i, and $O_{i}$ is the actual outcome for patient i. Brier score ranged from 0 to 1, and a Brier score of 0 indicates the best possible calibration. Sensitivity (recall), positive predictive value (PPV, precision), specificity, negative predictive value (NPV), and F1-score were also calculated as evaluation metrics. The R “pROC” and “PRROC” packages were used to calculate these metrics.

### 2.5 Detection of drug-drug interactions for risk of acute kidney injury

In the present study, the following two ML models were interpreted to detect interactions between two drug classes for increased risk of AKI: 1) the XGB model, which had the best predictive performance, and 2) the LLR model, which had the second-best predictive performance and can detect synergistic interactions on an additive scale. Although the complexity of the models of ML makes it hard to provide interpretability, some interpretation algorithms such as SHapley Additive exPlanations (SHAP) and Local Interpretable Model-Agnostic Explanations (LIME) have been used (Hu et al., 2022). In this study, SHAP values were calculated to detect features that affect the increased risk of AKI using the R “SHAPforxgboost” package.

In the LLR model, relative excess risk due to interaction (RERI) was used to evaluate synergistic interaction on an additive scale between two drug classes. RERI has been used to detect whether there are combined effects of two exposures on an outcome and can be calculated by substituting coefficients in the regression equation into the following Formulas 2, 3 (Knol et al., 2007; Knol and VanderWeele, 2012). RERI of 0 indicates no interaction on an additive scale. $\hat{\beta_{1}}$ , $\hat{\beta_{2}}$ , and $\hat{\beta_{3}}$ represent regression coefficients for drug class 1, drug class 2, and a product term of drug classes 1 and 2, respectively.

$$\left(e^{\hat{\beta_{1}}+\hat{\beta_{2}}+\hat{\beta_{3}}}-1\right)\neq \left(e^{\hat{\beta_{1}}}-1\right)+\left(e^{\hat{\beta_{2}}}-1\right)$$ (2)

and

$$RERI=e^{\hat{\beta_{1}}+\hat{\beta_{2}}+\hat{\beta_{3}}}-e^{\hat{\beta_{1}}}-e^{\hat{\beta_{2}}}+1$$ (3)

However, in the LLR model built using the glmnet package, a point estimate for each feature is calculated, but its standard error is not. Hence, 95% confidence intervals (95% CIs) of regression coefficients, adjusted odds ratio (OR), and RERI were estimated with a percentile bootstrap method (Figure 1) (Jung et al., 2019). To calculate 95% CIs, 2,000 bootstrap samples, each of which was the same size as the training set, were generated by resampling with replacement from the training set. After a parameter estimate was calculated from each bootstrap sample, 2,000 parameter estimates in all the bootstrap samples were sorted in ascending order. The interval between the 50th and 1950th quantile values of the 2,000 parameter estimates was regarded as the 95% CI. In this study, combinations that had a product term with a lower limit of adjusted OR 95% CI > 1 and had a lower limit of RERI 95% CI > 0 were considered to have a positive interaction for the risk of AKI. However, it is invalid to calculate RERI if the adjusted OR for at least one of two drug classes in a combination is less than 1.

### 2.6 Statistical analysis

To compare the patient characteristics between the case and control groups and between the training and testing sets, unpaired two-tailed Welch’s *t*-test or Wilcoxon rank-sum test for continuous data and chi-squared test for categorical data were performed. DeLong’s test and bootstrap test were performed to compare AUROC and AUPR, respectively. The level of statistical significance was set at 5.0% for all statistical analyses. All statistical analyses were performed using R software.

### 2.7 Sensitivity analyses

Sensitivity analyses were performed to evaluate the robustness of the detected potential drug-drug interactions. Since most cases of AKI occur within 4 weeks regardless of drug class, we redefined new exposures to the drug classes within 1–14 and 1–30 days from the event date as “use” and reconstructed the XGB and LLR models using the same procedure as above. SHAP and RERI values were calculated from the two reconstructed models, and potential interactions between two drug classes of interest were evaluated.

## 3 Results

### 3.1 Patients’ characteristics

A total of 65,667 eligible patients were extracted from NUSM’s CDW and assigned to the case (*N* = 5,319) and control (*N* = 60,348) groups. Their clinical characteristics are presented in Table 1. Age in the case group was significantly older than that in the control group (*p* < 0.001), and the case group contained significantly more male patients than the control group (*p* < 0.001). All seven medical diagnoses were significantly more prevalent in the case group than in the control group (all *p* < 0.001). With regard to therapeutic drug classes, most of the drug classes were significantly different between the case and control groups. SCr levels on the event date were within normal range (male, 0.65–1.07 mg/dL; female, 0.46–0.79 mg/dL) for both male and female subjects in the control group. On the other hand, most patients in the case group had SCr levels above the normal range, and SCr levels in the case group were significantly higher than those in the control group regardless of sex (*p* < 0.001, respectively). Additionally, patients’ characteristics were homogeneous between the training and testing sets in both the case and control groups (Supplementary Tables S6, S7).

**TABLE 1:** Patients’ characteristics in case and control groups.

Characteristics	Case group (N = 5,319)	Control group (N = 60,348)	*p* value
**Age (years), median (IQR)**	69 (59–78)	65 (51–74)	<0.001
**Male, n (%)**	3,519 (66.2)	32,726 (54.2)	<0.001
**Hospital, n (%)**			<0.001
Itabashi	3,924 (73.8)	43,998 (72.9)	
Surugadai	884 (16.6)	9,284 (15.4)	
Hikarigaoka	511 (9.6)	7,066 (11.7)	
**Medical history, n (%)**			
Hypertension	1,379 (25.9)	8,544 (14.2)	<0.001
Diabetes	1,602 (30.1)	13,994 (23.2)	<0.001
Heart failure	1,079 (20.3)	4,830 (8.0)	<0.001
Anemia	746 (14.0)	5,858 (9.7)	<0.001
Sepsis	620 (11.7)	1,107 (1.8)	<0.001
Chronic kidney disease	72 (1.4)	244 (0.4)	<0.001
Chronic liver disease	109 (2.0)	625 (1.0)	<0.001
**Use of therapeutic drug classes, n (%)**			
**Antibiotic drugs**			
Penicillins	462 (8.7)	2,731 (4.5)	<0.001
Cephalosporins	500 (9.4)	8,144 (13.5)	<0.001
Carbapenems	161 (3.0)	349 (0.6)	<0.001
Aminoglycosides	37 (0.7)	37 (0.1)	<0.001
Glycopeptides	228 (4.3)	86 (0.1)	<0.001
Tetracyclines	13 (0.2)	87 (0.1)	0.107
Fluoroquinolones	105 (2.0)	844 (1.4)	0.001
Macrolides	97 (1.8)	591 (1.0)	<0.001
Sulfamethoxazole/trimethoprim	58 (1.1)	104 (0.2)	<0.001
Azoles	21 (0.4)	29 (0.0)	<0.001
Amphotericin B	15 (0.3)	12 (0.0)	<0.001
Anti–herpes virus drugs (nucleoside analogues)	20 (0.4)	175 (0.3)	0.330
Interferons	3 (0.1)	31 (0.1)	1.000
** Antihypertensive drugs**			
Calcium channel blockers	495 (9.3)	4,628 (7.7)	<0.001
ACE inhibitors	225 (4.2)	2,164 (3.6)	0.018
ARBs	104 (2.0)	519 (0.9)	<0.001
α–adrenergic receptor blockers	35 (0.7)	185 (0.3)	<0.001
β–adrenergic receptor blocker	107 (2.0)	584 (1.0)	<0.001
Loop diuretics	516 (9.7)	1,170 (1.9)	<0.001
Aldosterone antagonists	246 (4.6)	658 (1.1)	<0.001
Other diuretics	24 (0.5)	277 (0.5)	1.000
** Antineoplastic drugs**			
Folate antimetabolites	16 (0.3)	11 (0.0)	<0.001
Platinum–based agents	133 (2.5)	125 (0.2)	<0.001
** Immunosuppressive drugs**			
Calcineurin inhibitors	8 (0.2)	23 (0.0)	0.001
Sulfhydryl compounds (DMARDs)	1 (0.0)	27 (0.0)	0.595
**Drugs for alimentary tract**			
Histamine H_2_ receptor blockers	637 (12.0)	7,391 (12.2)	0.577
Proton pump inhibitors	680 (12.8)	4,382 (7.3)	<0.001
**Drugs for dyslipidemia**			
Statins	142 (2.7)	1,966 (3.3)	0.022
Fibrates	11 (0.2)	203 (0.3)	0.143
**Others**			
NSAIDs	706 (13.3)	9,491 (15.7)	<0.001
SGLT2 inhibitors	5 (0.1)	52 (0.1)	1.000
Vitamin D_3_ preparations	2 (0.0)	100 (0.2)	0.036
**Serum creatinine on event date (mg/dL), median (IQR)**			
Male	1.7 (1.3–2.3)	0.8 (0.7–0.9)	<0.001
Female	1.3 (0.9–2.0)	0.6 (0.5–0.7)	<0.001

Since continuous data such as age and serum creatinine level were not normally distributed, Wilcoxon rank–sum test was performed for differences in the features. Chi–squared test was performed for categorical data. Abbreviations: DMARD, disease modifying anti–rheumatic drug; IQR, interquartile range; NSAID, non–steroidal anti–inflammatory drug; SGLT2, sodium glucose cotransporter 2.

### 3.2 Comparison of predictive performance among six ML models

**FIGURE 2:**
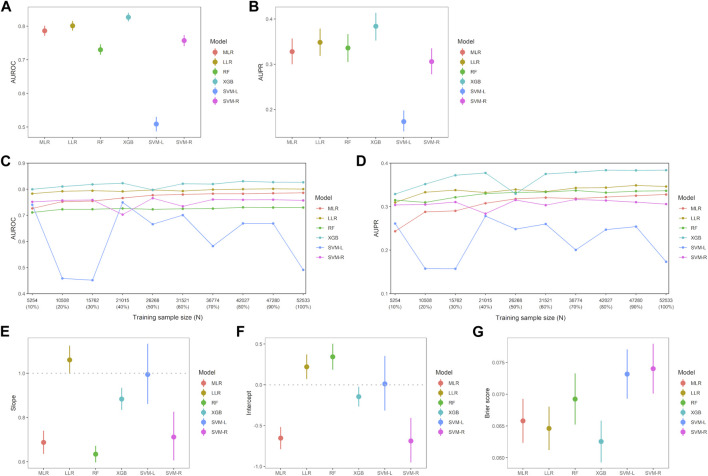
Comparison of evaluation metrics among six machine-learning models. **(A)** Each point indicates area under the receiver operating characteristic curve (AUROC) and error bar indicates 95% confidence interval (CI). There were significant differences between all six machine-learning models (*p* < 0.001, respectively). **(B)** Each point indicates area under the precision-recall curve (AUPR) and error bar indicates 95% CI. There were significant differences between all machine-learning models (*p* < 0.01, respectively) except between MLR and SVM-R models (*p* = 0.435), between MLR and RF models (*p* = 0.466), and between RF and LASSO model (*p* = 0.222). Robustness of the machine-learning models in AUROC **(C)** and AUPR **(D)**, respectively. AUROC for each training sample size was calculated by increasing the sample size by 10%. Calibration slope **(E)** and intercept **(F)** were calculated from the calibration curve, and error bar indicates 95% CI. **(G)** Brier score in each machine-learning model. Error bar indicates 95% CI and smaller Brier score indicates a stronger fit of the model. Abbreviations: LLR, logistic least absolute shrinkage and selection operator regression; MLR, multivariable logistic regression; RF, random forest; SVM-L, support vector machine (linear function); SVM-R, support vector machine (radial basis function); XGB, extreme gradient boosting.

Discrimination, robustness, and calibration of each ML model are shown in Figure 2, and other classification metrics are shown in Table 2. Among the six ML models, the XGB model had the highest AUROC (0.827, 95% CI 0.814–0.840), and the LLR model had the second highest AUROC (0.801, 0.787–0.816) (Figure 2A). The XGB model had the highest AUPR (0.384, 0.352–0.414) followed by the LLR (0.348, 0.319–0.379) and RF (0.336, 0.305–0.367) models (Figure 2B). The XGB model had the highest classification performance, with sensitivity of 77.4%, PPV of 20.5%, specificity of 72.9%, NPV of 97.3%, and F1-score of 0.324 (Table 2). After this model, the LLR model had sensitivity of 77.6%, PPV of 18.9%, specificity of 70.3%, NPV of 97.1%, and F1-score of 0.303. As for model robustness, the XGB and LLR models maintained high AUROC and AUPR even with small training sample sizes (10%–30% of the training set). The MLR model had low AUPR for very small training sample sizes, such as 10% of the training set, and the SVM-L model showed a lack of robustness (Figures 2C, D). With regard to model calibration, the LLR and SVM-L models had a good calibration slope (1.06, 1.00–1.12; 0.99, 0.86–1.13, respectively), and the latter also had a good calibration intercept (0.01, −0.32 to 0.35). On the other hand, the MLR, RF, XGB, and SVM-R models had slopes less than 1, and these three models other than the RF model had intercepts less than 0 (Figures 2E, F). The XGB model had the lowest Brier score (0.063, 0.059–0.066), followed by the LLR model (0.065, 0.061–0.068) (Figure 2G). Therefore, the XGB and LLR models, which had the best and second-best evaluation metrics, were interpreted to detect interactions between two drug classes with increased risk of AKI.

**TABLE 2:** Classification performance metrics of each machine-learning model.

	Machine-learning models					
Evaluation metrics	MLR	LLR	RF	XGB	SVM-L	SVM-R
Sensitivity (recall), %	72.2	76.6	64.8	77.4	46.5	67.6
PPV (precision), %	18.7	18.9	18.0	20.5	10.1	18.7
Specificity, %	71.7	70.3	73.3	72.9	62.6	73.5
NPV, %	96.6	97.1	95.8	97.3	92.8	96.2
F1-score	0.297	0.303	0.281	0.324	0.166	0.293
AUROC (95% CI)	0.786 [0.771, 0.802]	0.801 [0.787, 0.816]	0.730 [0.715, 0.746]	0.827 [0.814, 0.840]	0.509 [0.487, 0.530]	0.757 [0.741, 0.773]
AUPR (95% CI)	0.328 [0.300, 0.357]	0.348 [0.319, 0.379]	0.336 [0.305, 0.367]	0.384 [0.352, 0.414]	0.173 [0.151, 0.198]	0.306 [0.278, 0.335]

Abbreviations: AUPR, area under precision-recall curve; AUROC, area under receiver operating characteristic curve; CI, confidence interval; LLR, logistic least absolute shrinkage and selection operator regression; MLR, multiple logistic regression; NPV, negative predictive value; PPV, positive predictive value; RF, random forest; SVM-L, support vector machine with linear function kernel; SVM-R, support vector machine with radial basis function kernel; XGB, extreme gradient boosting.

Supplementary Figure S2 shows the effect of the 408 product terms on model discrimination of the six ML models. AUROC values of the five models, except for the MLR model, with these product terms were significantly higher than those of the models without them. AUPR values of the LLR, SVM-L, and SVM-R models with these product terms in the training set were significantly higher than those of the models without them. That is, the product terms between two drug classes affected model discrimination.

### 3.3 Evaluation of features affecting increased risk of AKI in XGB model

**FIGURE 3:**
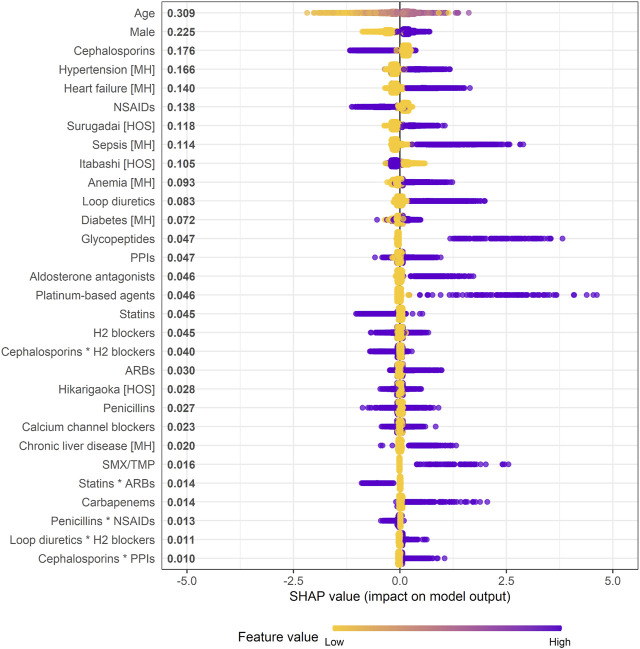
Effect of features on increased risk of AKI in XGB model (SHAP summary plot). * indicates product term of two drug classes. When a feature is continuous, the higher the feature value, the more purple it is. When a feature is binary, it is represented in purple if the feature is present. Each dot represents one patient on the line for each feature. Mean absolute SHAP value is shown to the right of a feature. Abbreviations: ARB, angiotensin receptor blocker; H_2_ blocker, histamine H_2_ receptor blocker; HOS, hospital; MH, medical history; NSAID, non-steroidal anti-inflammatory drug; PPI, proton pump inhibitor; SHAP, SHapley Additive exPlanation; SMX/TMP, sulfamethoxazole/trimethoprim.

A SHAP summary plot of the XGB model was made to identify features that affect the risk of AKI in the prediction model, and the top 30 important features are shown in Figure 3. All 110 features with non-zero mean (|SHAP|) values are shown in Supplementary Figure S3. This plot shows how strongly or weakly the features were related to the SHAP values. For example, the older the patient, the more purple it becomes. As another example, in the case of binary features such as sex and use or non-use of the drug classes, “male” and “use” are shown in purple. Age, male, hypertension, heart failure, and sepsis were detected as the most important predictors of increased risk of AKI, and their feature importance scores, as measured by mean (|SHAP|), were 0.309, 0.225, 0.166, 0.140, and 0.114, respectively. In particular, the locally-weighted scatterplot smoothing curve exceeded the SHAP value of 0 for ages from 60 to 93 years according to the SHAP dependence plot (Supplementary Figure S4). Regarding the individual drug classes, loop diuretics, glycopeptides, aldosterone antagonists, platinum-based agents, and sulfamethoxazole/trimethoprim were identified as important predictors of increased risk of AKI. Of the five product terms in the top 30 important features, the product term of loop diuretics and histamine H_2_ blockers [mean (|SHAP|) = 0.011], and that of cephalosporins and proton pump inhibitors (0.010) were identified as relatively important risk factors for AKI because SHAP values of most of the patients with these product terms were positive.

Supplementary Figure S5 shows the SHAP values of the reconstructed XGB models in which the drug classes newly started within 1–14 and 1–30 days were considered “use.” The product term of loop diuretics and H_2_ blockers was consistently included in the top 30 important features in the reconstructed models, and rather the SHAP values tended to be higher than those in the original model. Moreover, the individual effects of these drug classes on increased risk of AKI in the reconstructed models were also greater than those in the original model: mean (|SHAP|) of loop diuretics = 0.083 within 1–7 days, 0.116 within 1–14 days, and 0.161 within 1–30 days; that of H_2_ blockers = 0.045, 0.051, and 0.071, respectively.

### 3.4 Detection of drug-drug interactions for increased risk of AKI in LLR model

**FIGURE 4:**
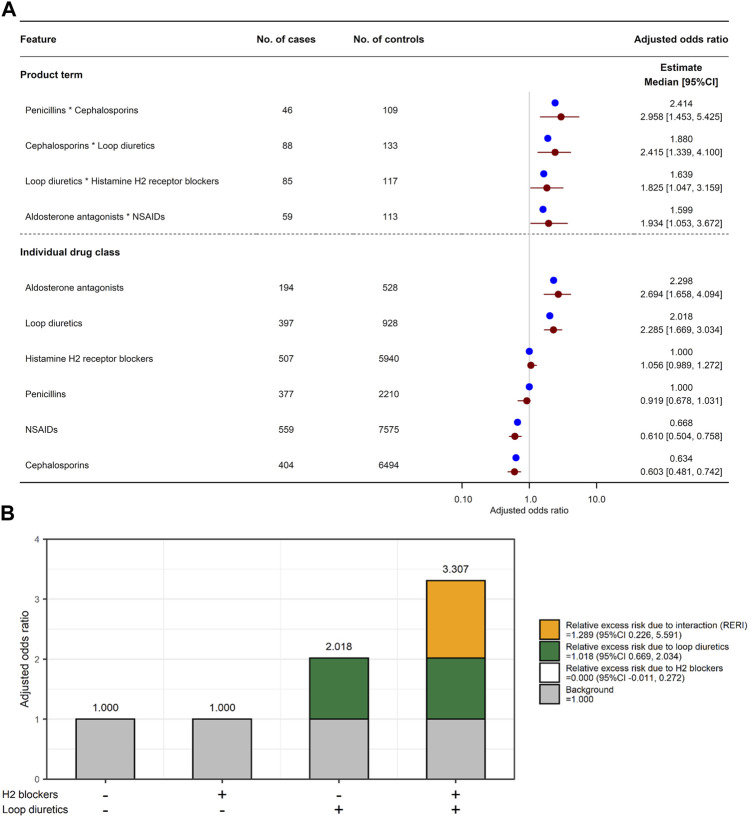
Combined effects of two drug classes on increased risk of AKI. **(A)** Adjusted odds ratio for six individual drug classes and their four product terms. Blue and red circles represent estimated adjusted odds ratio in the original training set and median of adjusted odds ratio in 2,000 bootstrap replicates, respectively. Red horizon indicates adjusted odds ratio 95% confidence interval calculated by a percentile bootstrap method. **(B)** Relative excess risk due to interaction (RERI) between histamine H_2_ blockers and loop diuretics. Gray bar indicates background (i.e., non-use of H_2_ blockers and loop diuretics). White and green bars indicate relative excess risk due to H_2_ blockers ( $e^{\hat{\beta_{1}}}-1$ ) and loop diuretics ( $e^{\hat{\beta_{2}}}-1$ ), respectively. Orange bar indicates RERI ( $e^{\hat{\beta_{1}}+\hat{\beta_{2}}+\hat{\beta_{3}}}-e^{\hat{\beta_{1}}}-e^{\hat{\beta_{2}}}+1$ ). Abbreviations: CI, confidence interval; NSAID, non-steroidal anti-inflammatory drug.

One hundred and thirty-four features were selected in the LLR model, with an optimized value *λ* of 0.0015275. All the selected features are shown in Supplementary Table S8. Of the 408 combinations, four combinations had a product term with a lower limit of adjusted OR 95% CI > 1 (Figure 4A): penicillins*cephalosporins (adjusted OR 2.414, 95% CI 1.453–5.425), cephalosporins*loop diuretics (1.880, 1.339–4.100), loop diuretics*H_2_ blockers (1.639, 1.047–3.159), and aldosterone antagonists*non-steroidal anti-inflammatory drugs (NSAIDs; 1.599, 1.053–3.672). Among the four combinations, only the combination of loop diuretics and histamine H_2_ blockers had a lower limit of RERI 95% CI > 0 (RERI 1.289, 95% CI 0.226 to 5.591 in Figure 4B): individual effect of loop diuretics, $e^{\hat{\beta_{1}}}$ = 2.018; that of histamine H_2_ blockers, $e^{\hat{\beta_{2}}}$ = 1.000; product term, $e^{\hat{\beta_{3}}}$ = 1.639; combined effect of these drug classes, $e^{\hat{\beta_{1}}+\hat{\beta_{2}}+\hat{\beta_{3}}}$ = 3.307. On the other hand, RERIs could not be calculated for the remaining three combinations because the adjusted ORs for the individual drug classes that were included in the product terms were less than 1 (e.g., NSAIDs; adjusted OR 0.668, 95% CI 0.504–0.758) (Knol and VanderWeele, 2012).

Supplementary Figure S6 shows the adjusted ORs for the four combinations of the six individual drug classes in the reconstructed LLR models. Similarly to the XGB model, the product term of loop diuretics and histamine H_2_ blockers and the individual effect of loop diuretics were consistently associated with increased risk of AKI in the reconstructed models. Moreover, exposure to H_2_ blockers within 1–30 days before the event date was significantly associated with increased risk of AKI (adjusted OR 1.485, 95% CI 1.089, 1.790).

## 4 Discussion

In the present study, six ML models were constructed for the prediction of AKI. Although the XGB model tended to overestimate the risk of AKI, this model had the best discrimination and the lowest Brier score among the six ML models. After the XGB model, the LLR model showed good discrimination and low Brier score. On the other hand, AUROC and AUPR of the SVM-L model were the lowest among these ML models. The reason for this is thought to be that the sample size was extremely large compared to the number of features, making it difficult to calculate a hyperplane that can clearly discriminate the presence or absence of AKI in the feature space. In fact, the SVM-R model, which maps the 452-dimensional feature space (input space) to a higher-dimensional feature space by using the radial basis function kernel, had significantly greater AUROC and AUPR than the SVM-L model. Therefore, we detected the combined effect of two therapeutic drug classes on increased risk of AKI by interpreting the XGB and LLR models, with good predictive performance.

In the XGB model, well-known risk factors for AKI such as older age (Xu et al., 2021), male sex (Neugarten, et al., 2018), and six medical diagnoses (Poston and Koyner, 2019; Ostermann et al., 2020; Yu et al., 2020; Cullaro et al., 2022) were included in the top 30 important predictors. Especially, the risk of AKI increased in Japanese elderly patients aged 60–93 years (Supplementary Figure S4). Similarly to the XGB model, age, male, and five diagnoses except for diabetes and chronic kidney disease were significantly associated with increased risk of AKI in the LLR model (Supplementary Table S8). Since life expectancy at birth in Japan is 81.1 years for men and 87.1 years for women (Tsugane, 2021), the range from 60 to 93 years associated with increased risk of AKI covers most of the Japanese elderly population; that is, Japanese elderly patients are at high risk of AKI. Regarding individual drug classes, five drug classes (loop diuretics, glycopeptides, aldosterone antagonists, platinum-based agents, and sulfamethoxazole/trimethoprim) were associated with increased risk of AKI in the XGB model. Furthermore, all of these drug classes associated with the risk of AKI in the XGB model were also significantly associated with the risk of AKI in the LLR model. The five drug classes associated with increased risk of AKI are known to be nephrotoxic drug classes (Usui et al., 2016; Nast, 2017; Ostermann et al., 2020). On the other hand, NSAIDs were associated with decreased risk of AKI in both the XGB and LLR models. Although NSAIDs are well-known risk factors for DIKD, recent studies suggest that the coexistence of other risk factors in patients who take NSAIDs contributes to the development of AKI. For example, the risk of NSAID-induced AKI in patients with CKD and elderly people tended to be higher than that in the general population (Zhang et al., 2017). Moreover, adding NSAIDs in patients with hypertension further increases blood pressure due to reduction of renal vasodilator prostanoids such as prostaglandin E_2_ (PGE_2_) and PGI_2_, which are formed predominantly by cyclooxygenase (COX)-2, leading to renal vascular damage (Drożdżal et al., 2021; Spence et al., 2022). Because these factors that modify the risk of NSAID-induced AKI were adjusted in this study, NSAIDs may not be associated with increased risk of AKI. Therefore, it is conceivable that the ML models constructed using electronic medical records can successfully explain the factors that affect the increased risk of AKI reported in various clinical studies to date.

In the LLR model, four product terms were significantly associated with increased risk of AKI. However, of these product terms, since three included a drug class with an adjusted OR <1 (e.g., NSAIDs and cephalosporins), RERI could not be calculated, suggesting that these three combinations are unlikely to have an interaction for the risk of AKI. In the XGB model, two product terms were identified as relatively important risk factors for AKI: loop diuretics * H_2_ blockers and cephalosporins * proton pump inhibitors. Although the product term of cephalosporins and proton pump inhibitors tended to be associated with increased risk of AKI, the individual effects of cephalosporins were suggested to reduce the risk, contrary to the product term. Therefore, this combination may not have an interaction for the risk of AKI. The product term of loop diuretics and H_2_ blockers was identified as an important predictor in both the XGB and LLR models, and the latter model suggested that concomitant use of these drug classes has a potential drug-drug interaction for AKI. Furthermore, the individual and combined effects of these drug classes on the risk of AKI were robust in the sensitivity analyses. To our knowledge, no clinical studies have evaluated the association between H_2_ blockers and AKI in a large population, but Fisher et al. have summarized more than 20 case reports of H_2_ blocker-induced AIN (Fisher and Le Couteur, 2001). Since drug-induced AIN is a common cause of AKI (Perazella and Markowitz, 2010), it is not surprising that H_2_ blockers are one of the risk factors for AKI. According to the drug-drug interaction checker by DrugBank, the combination of loop diuretic and H_2_ blocker is suggested to have a drug-drug interaction that affects organic anion transporter 3 (OAT3), and its severity is moderate. SCr is excreted into urine through renal drug transporters such as OAT2, organic cation transporter 2 (OCT2), OCT3, multidrug and toxin extrusion protein 1 (MATE1), and MATE2-K (Nakada et al., 2019). Loop diuretics including furosemide and torasemide are known to be human OAT1 (hOAT1), hOAT3, and hOAT4 inhibitors (Vormfelde et al., 2006; Jeong et al., 2015; Gharibkandi et al., 2022), and it has been reported that uptake of H_2_ blockers such as famotidine and cimetidine into hOAT3-expressing cells decreases in the presence of an hOAT3 inhibitor (Tahara et al., 2005). That is, concomitant use of loop diuretics, which are OAT inhibitors, with H_2_ blockers may increase the concentration of H_2_ blockers in the blood. H_2_ blockers including famotidine, cimetidine, and nizatidine are known as *in vitro* OAT2-, OCT2-, OCT3-, MATE1-, and MATE2-K-inhibitors, and these drugs increase SCr in healthy subjects (Nakada et al., 2019). For these reasons, we speculate that loop diuretics reduce renal excretion of H_2_ blockers, and then OATs and OCTs expressed at the basolateral membrane of proximal tubule cells of the human kidney are inhibited by these drugs, resulting in elevated SCr. Therefore, although the relative importance of the individual and combined effects of loop diuretics and H_2_ blockers in the XGB model was lower than that of well-known risk factors such as older age, sex, and medical history, it was suggested that the interaction between loop diuretics and H_2_ blockers can increase the risk of AKI.

The present study has several limitations. First, there is a possibility of sampling bias because this study was a case-control study design using non-randomized data. Second, this study controlled potential confounding factors that were available and measurable, but failed to adjust for non-observed risk factors. For example, AKI is a common complication after cardiac surgery, and percutaneous coronary intervention is a known risk factor for AKI, and the incidence of cardiac surgery-induced AKI in Japanese patients is similar to that in other countries (Marenzi et al., 2013; Karrowni et al., 2016; Ikemura et al., 2020). However, no surgical information is recorded in NUSM’s CDW. As another example, acute physiologic assessment and chronic health evaluation (APACHE) Ⅱ, which is a scoring system for assessing the severity of ICU inpatients, is a risk factor for AKI in patients with severe sepsis (Chawla et al., 2007). Unfortunately, since there are no APACHE 2 scores recorded in our database, it is very difficult to adjust for APACHE II score as a covariate in this study. Finally, the database cannot access clinical information stored at other medical institutions. In this study, drug classes that were newly started within 1–7 days from the event date in the three hospitals were considered as suspected drug classes for AKI, but the drug classes may have been previously prescribed by another medical institution. However, the two ML models showed better predictive performance than the traditional statistical model, and the clear drug-drug interactions detected by interpreting these models may be useful for drug prescribing decision making.

## Data Availability

The raw data supporting the conclusion of this article will be made available by the authors, without undue reservation.
