# Supplementary material for: Detection of potential drug-drug interactions for risk of acute kidney injury: a population-based case-control study using interpretable machine-learning models
Source: Front Pharmacol. 2023 May 23;14:1176096. doi: 10.3389/fphar.2023.1176096 (PMC10242015; doi:10.3389/fphar.2023.1176096)
Supplement: Supplementary file 1 [file DataSheet1.pdf]

*Supplementary Figures*

# Detection of potential drug-drug interactions for risk of acute kidney injury: A population-based case-control study using interpretable machine-learning models

**Hayato Akimoto[1,2]*, Takashi Hayakawa[1,2], Takuya Nagashima[1,2], Kimino Minagawa[2], Yasuo Takahashi[2], Satoshi Asai[1,2]**

*** Correspondence:** Hayato Akimoto: akimoto.hayato@nihon-u.ac.jp

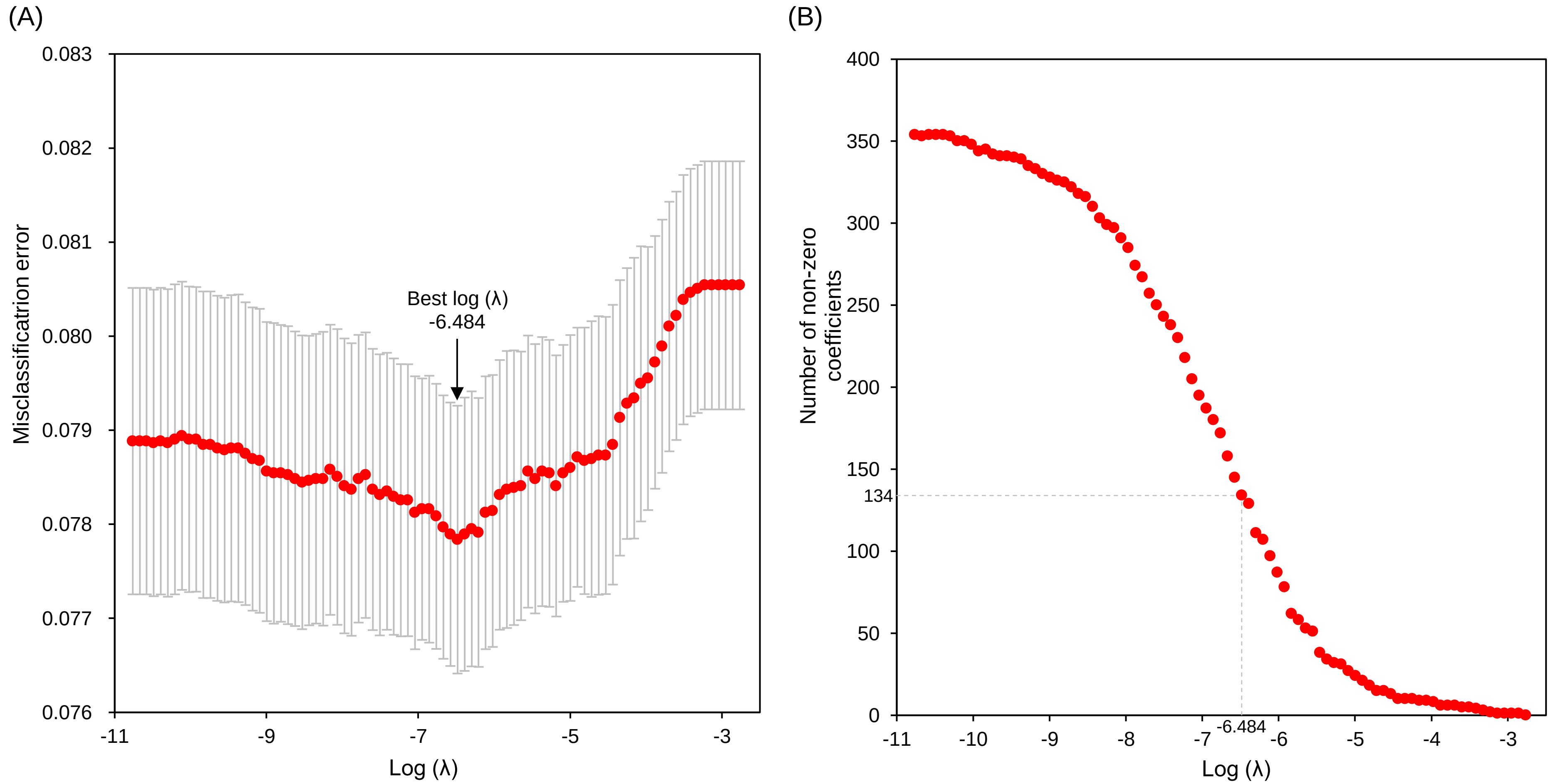


***Supplementary Figure S1. Feature selection in logistic least absolute shrinkage and selection operator (LLR) model***. (A) Ten-fold cross validation plot for the penalty term lambda (λ). Error bar indicates standard deviation for misclassification error in 10 iterations. A larger λ results in more coefficients being forced to be zero. For this hyperparameter tuning in the LLR model, λ values ranged from 0.0000211 to 0.0631024, with the minimum misclassification error rate achieved as 0.0015275 (Log (λ) = -6.484). (B) Number of non-zero regression coefficients for each λ value. The LLR model included 134 non-zero regression coefficients for the best log (λ).

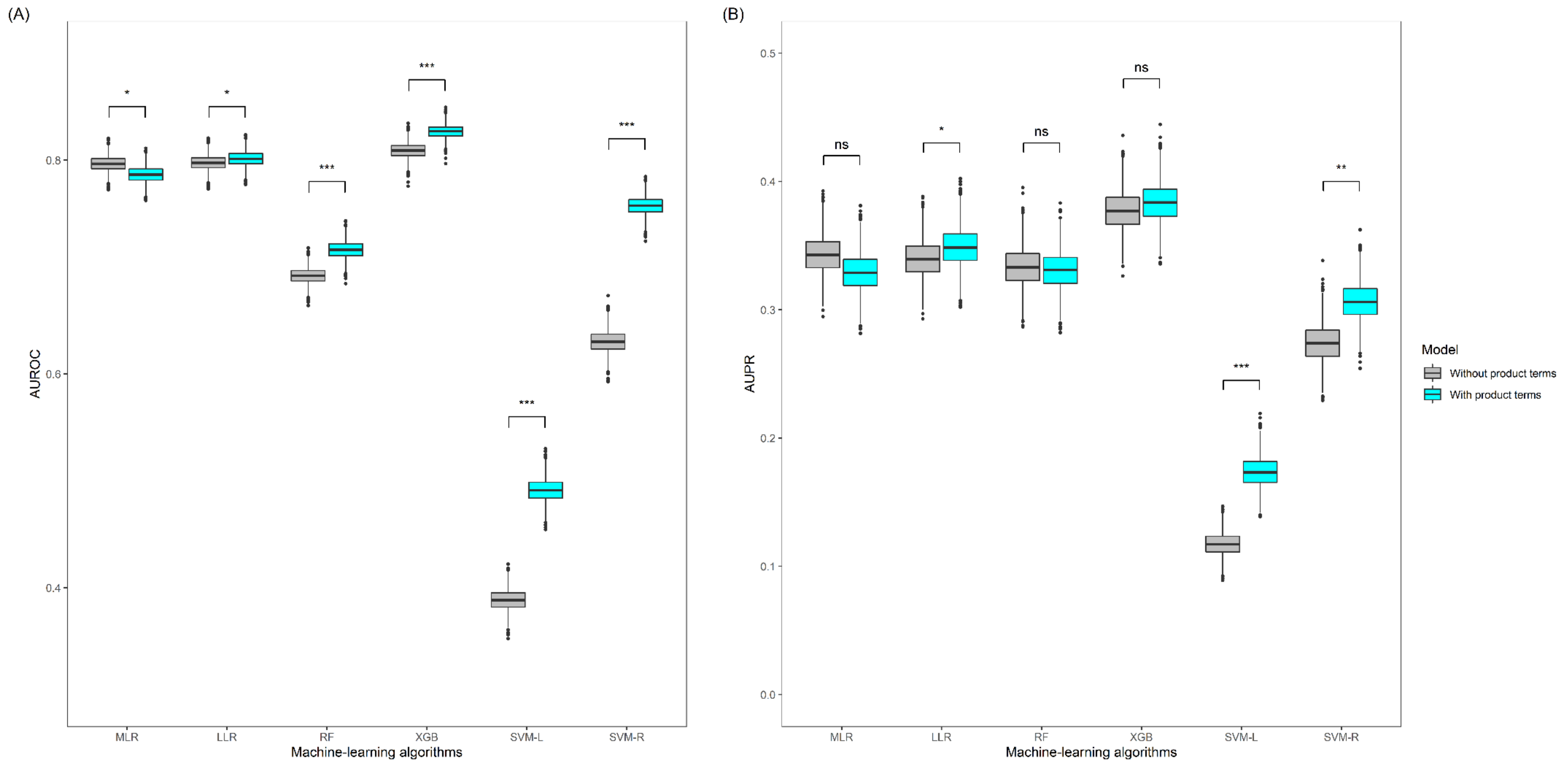


***Supplementary Figure S2. Effect of product terms on evaluation metrics in each machine-learning algorithm.*** (A) Comparison of area under the receiver operating characteristic curve (AUROC) between presence (light blue) or absence (gray) of interaction terms included in dataset. (B) Comparison of area under the precision-recall curve (AUPR) between presence (light blue) or absence (gray) of interaction terms. * $p < 0.05$, ** $p < 0.01$, *** $p < 0.001$. Abbreviations: LLR, logistic least absolute shrinkage and selection operator regression; MLR, multivariable logistic regression; ns, not significant; RF, random forest; SVM-L, support vector machine (linear function); SVM-R, support vector machine (radial basis function); XGB, extreme gradient boosting.

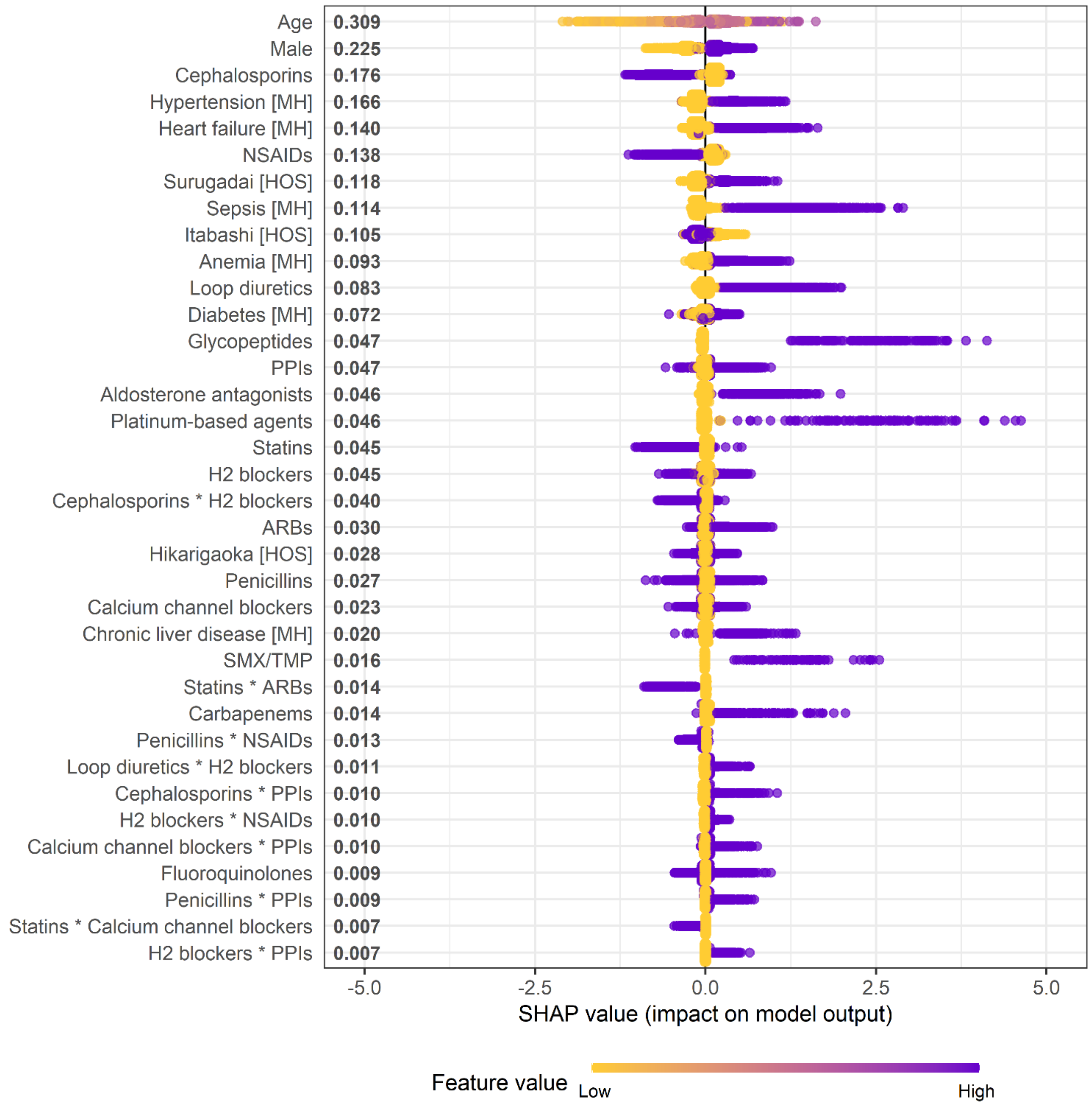


***Supplementary Figure S3. All 110 features with non-zero mean absolute SHapley Additive exPlanations (SHAP) values.*** Abbreviations: ACE, angiotensin converting enzyme; ARB, angiotensin receptor blocker; DMARD, disease modified anti-rheumatic drug; HOS, hospital; MH, medical history; NSAID, non-steroidal anti-inflammatory drug; PPI, proton pump inhibitor; SMX/TMP, sulfamethoxazole/trimethoprim.

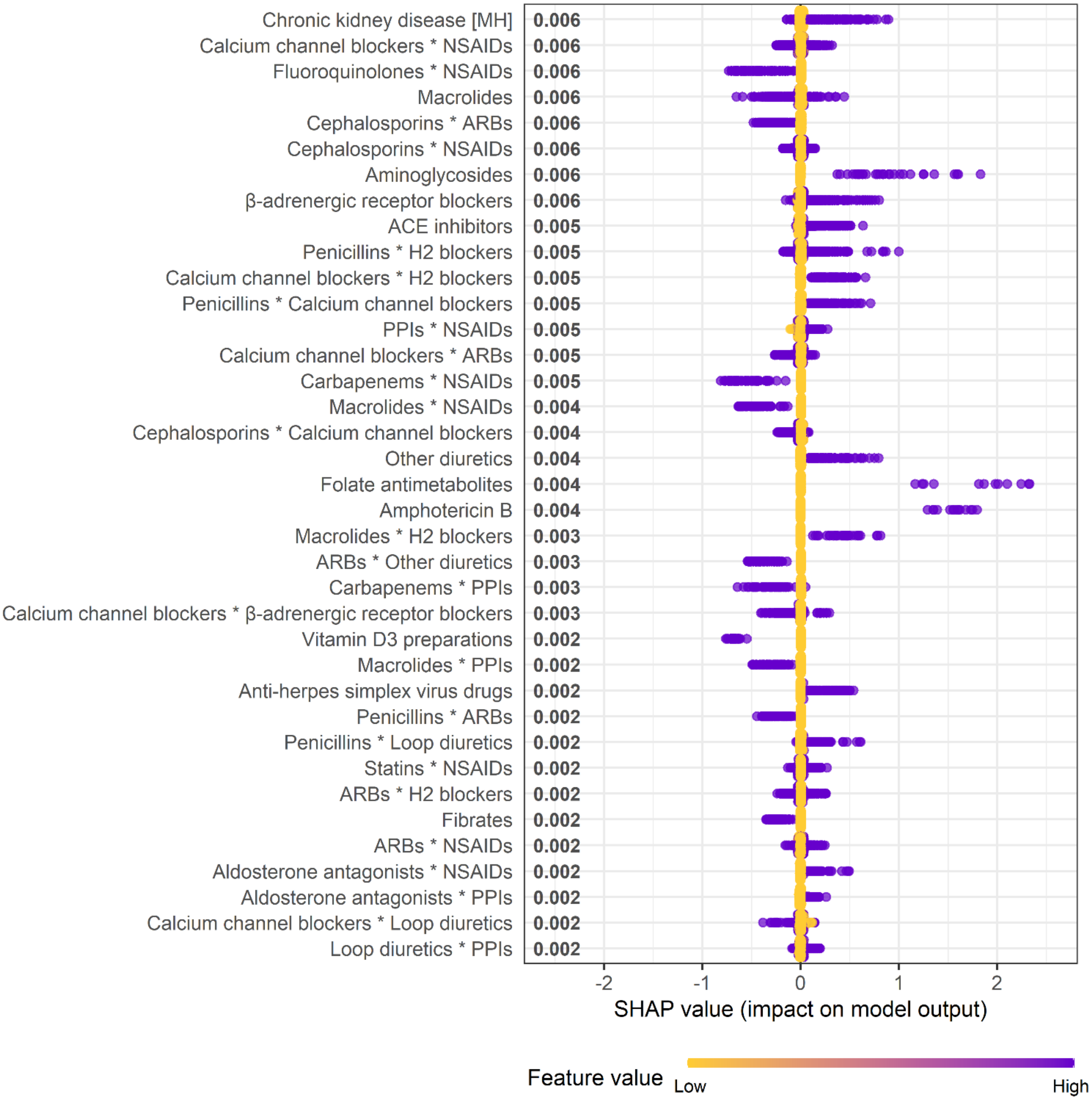


***Supplementary Figure S3 (continued). All 110 features with non-zero mean absolute SHapley Additive exPlanations (SHAP) values.*** Abbreviations: ACE, angiotensin converting enzyme; ARB, angiotensin receptor blocker; DMARD, disease modified anti-rheumatic drug; HOS, hospital; MH, medical history; NSAID, non-steroidal anti-inflammatory drug; PPI, proton pump inhibitor; SMX/TMP, sulfamethoxazole/trimethoprim.

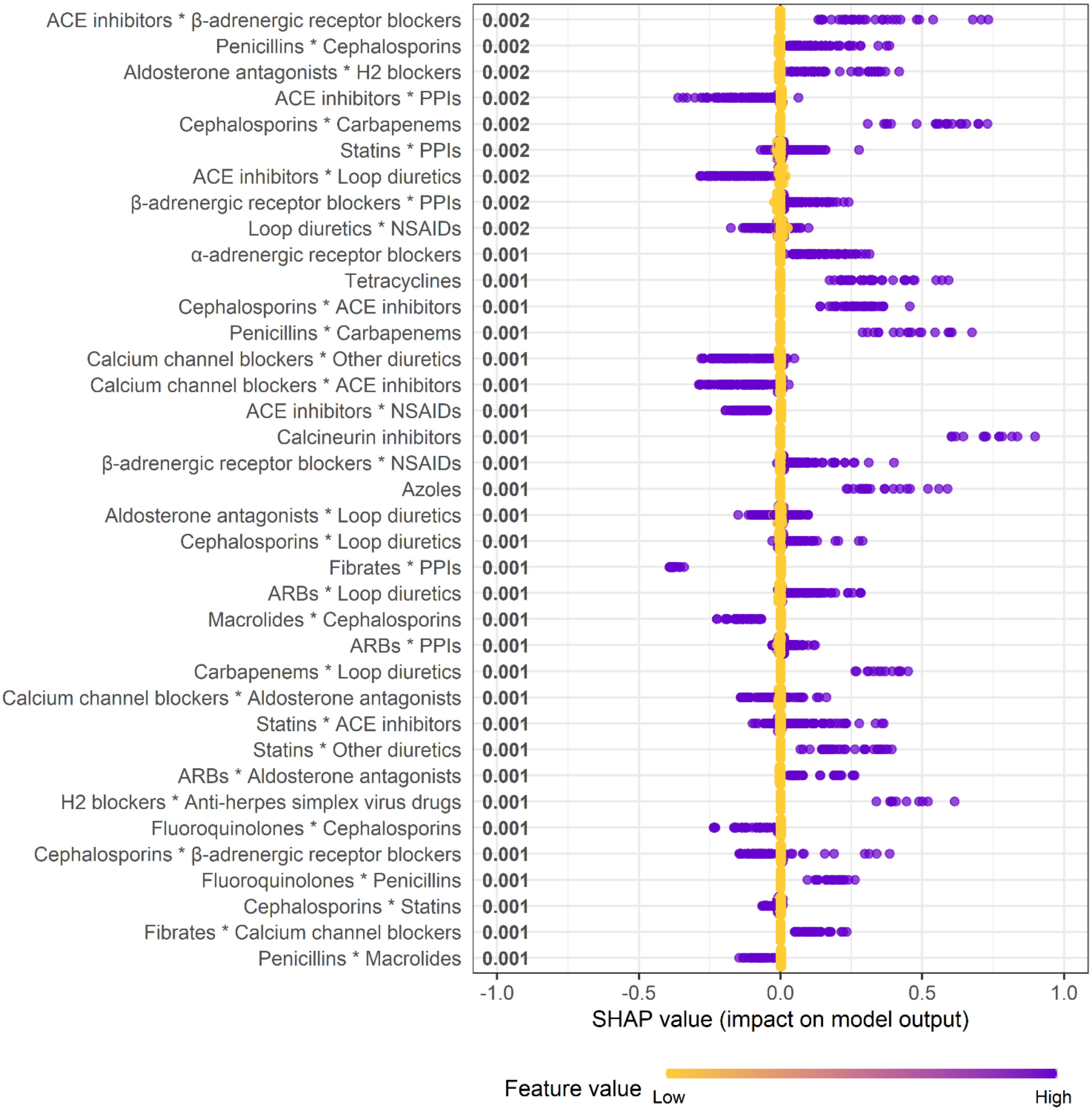


***Supplementary Figure S3 (continued). All 110 features with non-zero mean absolute SHapley Additive exPlanations (SHAP) values.*** Abbreviations: ACE, angiotensin converting enzyme; ARB, angiotensin receptor blocker; DMARD, disease modified anti-rheumatic drug; HOS, hospital; MH, medical history; NSAID, non-steroidal anti-inflammatory drug; PPI, proton pump inhibitor; SMX/TMP, sulfamethoxazole/trimethoprim.

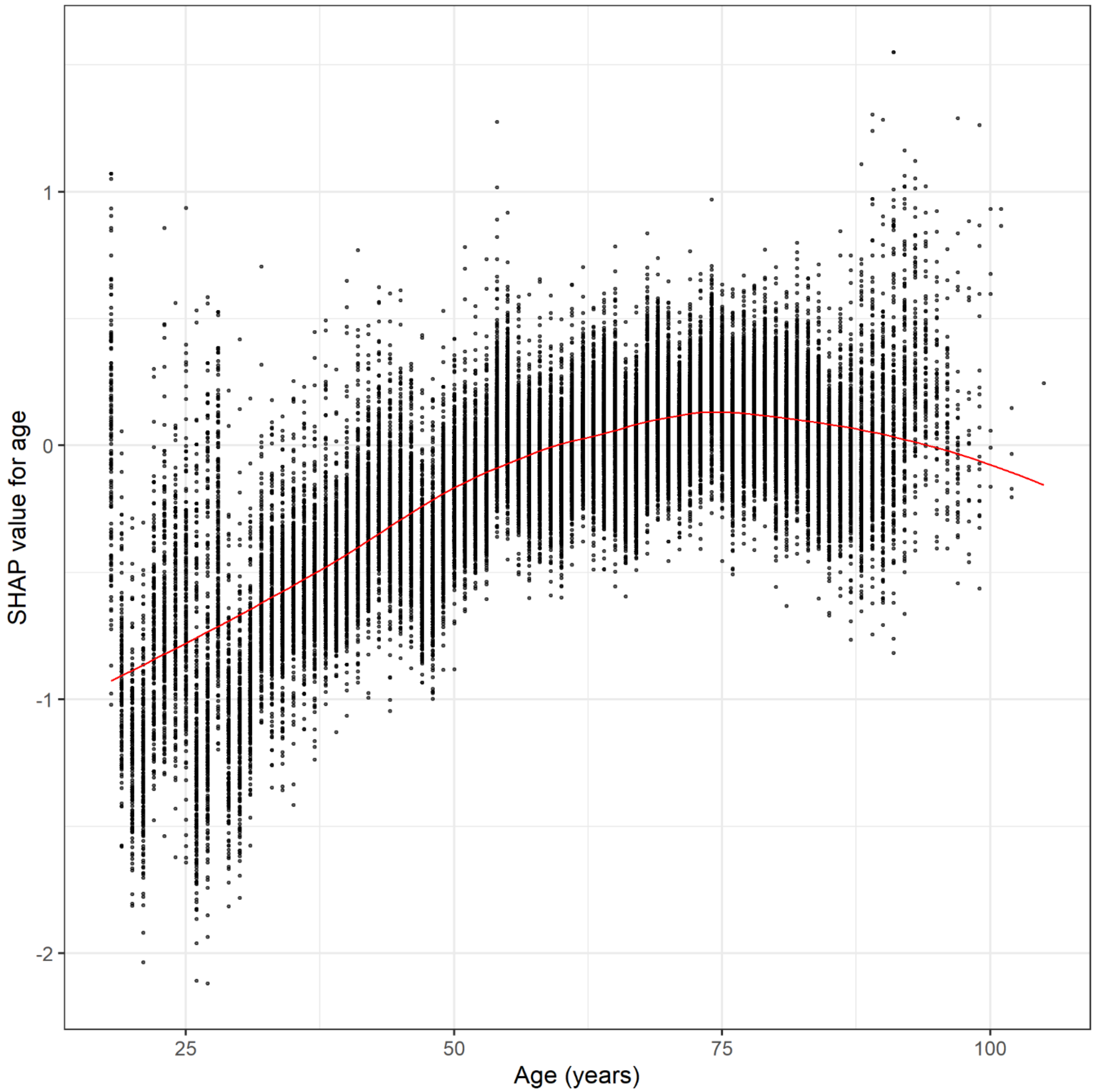


***Supplementary Figure S4. SHapley Additive exPlanations (SHAP) dependence plot for age in extreme gradient boosting (XGB) model.*** SHAP values exceeding 0 represent an increased risk of acute kidney injury. Red line indicates locally estimated scatterplot smoothing curve.

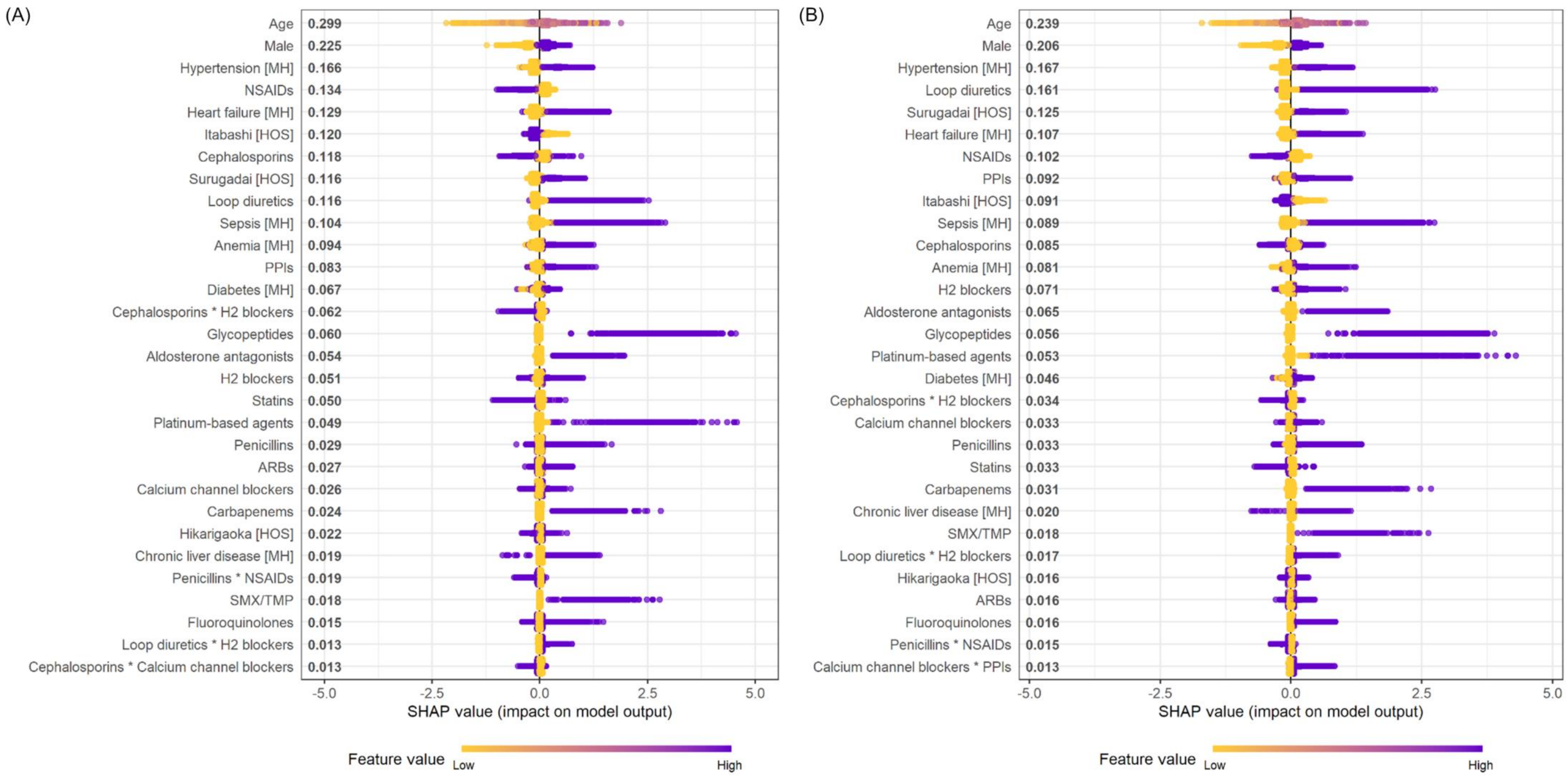


***Supplementary Figure S5. Sensitivity analyses in extreme gradient boosting (XGB) model.*** (A) SHAP summary plot of reconstructed XGB model in which newly started drug classes within 1-14 days from the event date were considered "use". (B) SHAP summary plot in which newly started drug classes within 1-30 days from the event date were considered "use". Abbreviations: ARB, angiotensin receptor blocker; H2 blocker, histamine $H_2$ receptor blocker; HOS, hospital; MH, medical history; NSAID, non-steroidal anti-inflammatory drug; PPI, proton pump inhibitor; SHAP, SHapley Additive exPlanation; SMX/TMP, sulfamethoxazole/trimethoprim.

| Feature | No. of cases | No. of controls | Adjusted odds ratio Estimate | Adjusted odds ratio Median [95%CI] |
|---|---|---|---|---|
| **Product term** | | | | |
| Penicillins * Cephalosporins | | | | |
| within 1-14 days | 93 | 139 | 2.051 | 2.800 [1.234, 4.848] |
| within 1-30 days | 168 | 205 | 2.800 | 3.063 [1.658, 4.788] |
| Cephalosporins * Loop diuretics | | | | |
| within 1-14 days | 171 | 155 | 1.756 | 2.272 [1.188, 3.573] |
| within 1-30 days | 294 | 191 | 2.156 | 2.351 [1.495, 3.448] |
| Loop diuretics * Histamine H2 receptor blockers | | | | |
| within 1-14 days | 156 | 141 | 1.429 | 1.692 [1.009, 2.653] |
| within 1-30 days | 249 | 170 | 1.582 | 1.662 [1.149, 2.474] |
| Aldosterone antagonists * NSAIDs | | | | |
| within 1-14 days | 92 | 142 | 1.360 | 1.570 [1.000, 2.780] |
| within 1-30 days | 163 | 158 | 1.422 | 1.458 [1.000, 2.318] |
| **Individual drug class** | | | | |
| Aldosterone antagonists | | | | |
| within 1-14 days | 286 | 557 | 2.282 | 2.929 [1.745, 4.468] |
| within 1-30 days | 393 | 610 | 3.028 | 3.191 [2.025, 4.718] |
| Loop diuretics | | | | |
| within 1-14 days | 616 | 1042 | 2.409 | 2.736 [2.084, 3.594] |
| within 1-30 days | 818 | 1147 | 2.860 | 2.852 [2.221, 3.669] |
| Histamine H2 receptor blockers | | | | |
| within 1-14 days | 694 | 6341 | 1.124 | 1.272 [1.000, 1.530] |
| within 1-30 days | 913 | 6736 | 1.485 | 1.498 [1.089, 1.790] |
| Penicillins | | | | |
| within 1-14 days | 522 | 2250 | 1.000 | 1.000 [0.865, 1.208] |
| within 1-30 days | 680 | 2406 | 1.035 | 1.012 [0.846, 1.282] |
| NSAIDs | | | | |
| within 1-14 days | 791 | 8368 | 0.779 | 0.701 [0.584, 0.903] |
| within 1-30 days | 1045 | 9372 | 0.791 | 0.773 [0.651, 0.950] |
| Cephalosporins | | | | |
| within 1-14 days | 623 | 6931 | 0.867 | 0.834 [0.682, 1.000] |
| within 1-30 days | 860 | 7539 | 0.887 | 0.863 [0.704, 1.000] |

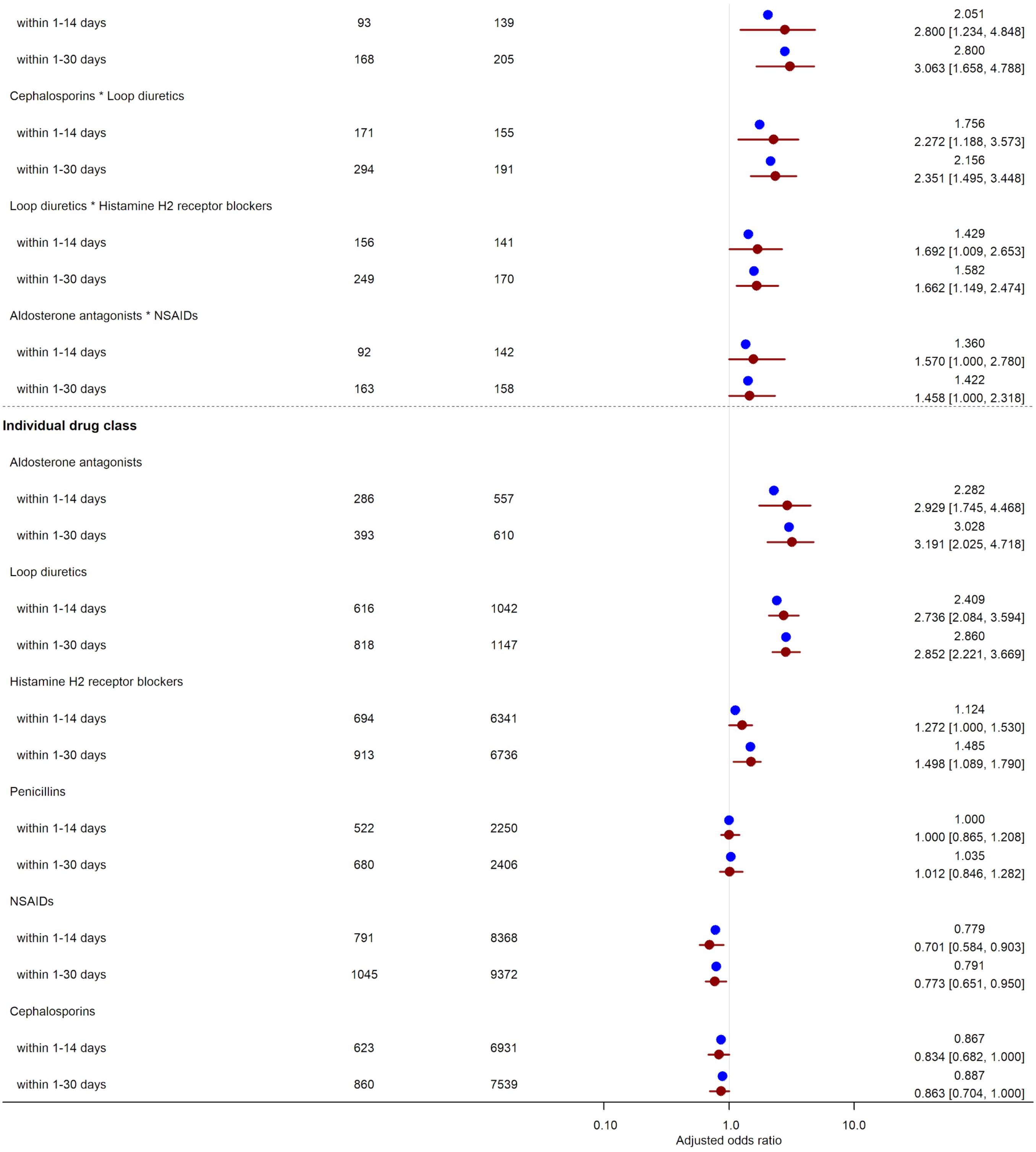

***Supplementary Figure S6. Sensitivity analyses in logistic least absolute shrinkage and selection operator regression (LLR) model.*** Adjusted odds ratio for four product terms of six drug classes in the two reconstructed LLR models. Abbreviations: CI, confidence interval; NSAID, non-steroidal anti-inflammatory drug.
